# Identify clinical factors related to Mycoplasma pneumoniae pneumonia with hypoxia in children

**DOI:** 10.1186/s12879-020-05270-6

**Published:** 2020-07-22

**Authors:** Yaoyao Ling, Tongqiang Zhang, Wei Guo, Zhenli Zhu, Jiao Tian, Chunquan Cai, Yongsheng Xu

**Affiliations:** 1 Tianjin Medical University, No.22, Qixiangtai Road, Heping District, Tianjin, 300070 China; 2 Department of Respiratory, The Children’s Hospital of Tianjin (Children’s Hospital of Tianjin University), Tianjin, China; 3 Department of Neurosurgery, The Children’s Hospital of Tianjin (Children’s Hospital of Tianjin University), Tianjin, China

**Keywords:** Mycoplasma pneumoniae pneumonia, Hypoxia, Clinical factors, Children

## Abstract

**Background:**

To analyze the clinical characteristics of Mycoplasma pneumoniae pneumonia with hypoxia in children, and identify the associated risk factors of hypoxia in MPP.

**Methods:**

A retrospective case-control study was performed on 345 children with Mycoplasma pneumoniae pneumonia (MPP) admitted to our hospital wards from January 2017 to June 2019. They were divided into three groups, namely MPP with hypoxia, refractory Mycoplasma pneumoniae pneumonia (RMPP), and general Mycoplasma pneumoniae pneumonia (GMPP). The clinical features, laboratory findings, imaging, and management were collected and compared in the three groups.

**Results:**

The MPP with hypoxia patients (*n* = 69) had longer disease duration, a higher extra-pulmonary complications rate, and more severe radiological abnormalities (*P* < 0.05). They also needed more complicated treatments (*P* < 0.05). Meanwhile, the levels of white blood cell count (WBC), C-reactive protein (CRP), lactic dehydrogenase (LDH), interleukin (IL)-6, ferritin, D-dimer, fibrinogen (FG), alanine aminotransferase (ALT) and the percentage of neutrophils in the MPP with hypoxia group were significantly higher than those in the RMPP group and the GMPP group (*P* < 0.05). In ROC curve analysis, the percentage of neutrophils, WBC, CRP, LDH, IL-6, ferritin, D-dimer, and ALT were contributed to identify the MPP with hypoxia patients. Multivariate logistic regression analysis revealed that ferritin> 174.15 ng/mL, IL-6 > 25.475 pg/ml, and pleural effusion were significantly associated with the incidence of hypoxia in MPP (*P* < 0.01).

**Conclusion:**

MPP with hypoxia patients presented more serious clinical manifestations. Ferritin> 174.15 ng/mL, IL-6 > 25.475 pg/ml and pleural effusion were related risk factors for hypoxia in MPP.

## Background

Mycoplasma pneumoniae pneumonia (MPP) is one of the commonest causes of pediatric community-acquired pneumonia, causing 10–40%of cases [1, 2]. MPP, often described as a self-limiting disease, is typically mild and cured without medication [3], but sometimes it can develop into a severe and/or fulminant disease, which is always with severe complications such as respiratory failure, hypoxia, and even acute respiratory distress syndrome (ARDS) [4, 5]. As we all know, hypoxia often relates to rapid disease progression and death. Therefore, it is important for clinicians to recognize the MPP with hypoxia earlier, grasp the appropriate opportunity for reasonable therapy, and reduce complications.

In our study, we retrospectively analyzed the cases of MPP hospitalized in our hospital between January 2017 and June 2019. Then to explore the related factors predicting MPP with hypoxia, we compared the differences of clinical features, laboratory findings, imaging, and treatments in the MPP with hypoxia, refractory Mycoplasma pneumoniae pneumonia (RMPP), and general Mycoplasma pneumoniae pneumonia (GMPP).

## Methods

### Patient selection

#### Clinical information

Sixty-nine patients in MPP with hypoxia were admitted to the Respiratory Department of Tianjin Children’s Hospital from January 2017 to June 2019. We also randomly selected 86 patients in the RMPP group and 190 patients in the GMPP group from the same period. All cases met the diagnostic criteria.

#### Diagnostic criteria

All patients had clinical evidence of pneumonia on admission such as a fever, cough, and pneumonic infiltrations in the chest radiograph. *MP* infection was based on the positive results for *MP* polymerase chain reaction (PCR) tests of nasopharyngeal secretions (88.70%) or positive results of a serological test (11.3%). Anti-MP IgM titrations were performed twice at the time of admission and before discharge, and we selected patients whose test result was a seroconversion (negative to positive), four-fold or greater increase in IgM titers, or both high titers of>1:640 (MP-IgM antibody titer ≥1:160) [6]. Hypoxia was defined as any recorded oxygen saturation of < 92% by pulse oximetry, measured on room air [7]. RMPP mainly referred to the MPP characterized by persistent fever and progressive exacerbations of clinical symptoms, signs, and related imaging manifestations after standard treatment with macrolide drugs for ≥1 week [8].

#### The inclusion criteria

(1) met the diagnostic criteria; (2) the age was less than 15 years old.

#### The exclusion criteria

(1) someone had other respiratory pathogen infections and tuberculosis by following tests: blood cultures, nasopharyngeal aspirate cultures, nasopharyngeal aspirate for virus reverse transcriptase real-time multiplex PCR, serology for Chlamydia pneumoniae (CT) and *Legionella pneumophila* (LG), and protein purified derivative (PPD). (2) someone had basic diseases such as asthma, chronic cardiopulmonary disease, rheumatism and immune deficiency. (3) someone had a previous history of hypoxia. (4) someone had used glucocorticoid before admission.

#### Data collection

Hospitalization demographic, clinical information, laboratory findings, imaging, and management of all children included in the study were collected retrospectively. Nasopharyngeal aspirate specimens were routinely collected within 24 h of admission.

Respiratory specimens were tested for bacterial culture, virus using RT-mPCR, and *MP* using PCR. Peripheral blood samples were obtained on admission for the determination of complete blood count, C-reactive protein (CRP), lactic dehydrogenase (LDH), procalcitonin (PCT), interleukin (IL)-6, lactic acid, ferritin (Fer), D-dimer, fibrinogen (Fg), alanine aminotransferase (ALT), aspartate aminotransferase (AST) and specific antibody to *MP*. Blood culture was also performed on admission. Chest radiography was performed before admission or during hospitalization. If a patient had one of the following conditions, he or she would undergo a CT scan: 1. the clinical manifestations inconsistent with the chest radiograph;2. suspected airway and lung malformations; 3.serious complications associated with pneumonia; 4.routine treatment ineffective, and exclude other diseases such as interstitial lung disease, pulmonary tuberculosis, so on [9, 10]. The percent of CT scans in MPP with hypoxia, RMPP and GMPP was 100,84.88, and 17.69% respectively.

#### Observation indexes

Clinical features (sex, age, duration of fever, peak fever, dyspnea, complications, etc.), laboratory findings, imaging, hospitalization time, and treatments.

#### Ethics

The study was approved by the ethics committee of the Tianjin Children’s Hospital. And the data from patients were analyzed anonymously.

#### Data analysis

SPSS 22.0 was used for statistical analysis. The normal distribution data was represented by mean ± SD ($\overline{x}\pm s$). One-way ANOVA was used for comparison between groups. The LSDt-test was used for comparison within the group. The skewed distribution data were expressed as median (P25, P75), which comparisons were made by the Mann-Whitney U-test. And Chi-squared tests were used to compare categorical data. Receiver operating characteristic (ROC) curves were operated to evaluate candidate markers related to MPP with hypoxia, and logistic regression analysis was performed to select variables associated with MPP with hypoxia. The difference was considered statistically significant at *P* < 0.05.

## Results

### Basic information of patients

One hundred ninety-four male and 151 female patients with a median age of 8 (4–6) years were included in this study. There were 69 cases in the MPP with hypoxia group (34 men; 35 women),86 cases in the RMPP group (48 men; 38women), and 190 cases in the GMPP group (112 men; 78 women). The median age was 6 (4–8) years in the MPP with hypoxia,6 (4–8) years in the RMPP, and 6 (4–7) years in GMPP. There was no statistical difference in age and gender among the three groups.

### Clinical characteristics of patients (Table 1)

All patients presented a cough, and 350(98.87%) patients had fever. And the MPP with hypoxia group had a higher fever (39.1–41 °C) than the other two groups (*P* < 0.05). Moreover, differences were observed in the incidence of rash, liver function damage, chest pain, toxic encephalopathy, thromboembolism, dyspnea, mucous plugging between the MPP with hypoxia and the other two groups (*P* < 0.05).

**Table 1:** Clinical characteristic of MPP with hypoxia, RMPP and GMPP patients

Clinical information	MPP WITH HYPOXIA(***n*** = 69)	RMPP(***n*** = 86)	GMPP(***n*** = 190)	***P***-value
Sex (male/female)	34/35	48/38	112/78	0.381
Age, years	6 (4–8)	6 (4–8)	6 (4–7)	0.125
Clinical presentation n(%)				
Fever	69 (100%)	86 (100%)	186 (97.89%)	1.000
				0.000
37.5–38 °C	0 (0%)	0 (0%)	10 (5.38%)	
38.1–39 °C	6 (8.69%)	20 (23.25%)	53 (28.49%)	
39.1–41 °C	61 (88.41%)	64 (74.42%)	122 (65.59%)	
>41 °C	2 (2.90%)	2 (2.33%)	1 (0.54%)	
Cough	69 (100%)	86 (100%)	190 (100%)	1.000
Chest pain	18 (26.09%)	3 (3.49%)	2 (1.05%)	0.000
Rash	10 (14.49%)	3 (3.49%)	14 (7.37%)	0.043
Thromboembolism	7 (10.14%)	0 (0%)	0 (0%)	0.000
Wheezing	8 (11.59%)	10 (11.63%)	30 (15.79%)	0.533
Dyspnea	69 (100%)	2 (2.32%)	0 (0%)	0.000
Liver function damage	13 (18.84%)	5 (5.81%)	16 (8.42%)	0.023
Toxic encephalopathy	9 (13.04%)	2 (2.32%)	0 (0%)	0.000
Mucous plugging	36 (52.17%)	16 (18.60%)	2 (1.05%)	0.000
Length of fever, days	12 (9–14)	10 (8–12)	6 (8–10)	0.000
Length of stay, days	12 (9–15)	9 (8–10)	6 (5–7)	0.000
Management				
Using Azithromycin	69 (100%)	86 (100%)	130 (100%)	1.000
Using glucocorticoids	69 (100%)	58 (84.06%)	78 (41.05%)	0.000
Using gamma immunoglobulin	34 (49.27%)	0 (0%)	0 (0%)	0.000
Using fiberoptic bronchoscope	58 (84.06%)	61 (70.93%)	82 (43.16%)	0.000
Using oxygen-therapy	69 (100%)	2 (2.32%)	0 (0%)	0.000

Data are presented as number (percentage), median (25th–75th percentile)

### Laboratory and imagine the features of patients

Laboratory findings and imaging manifestations in the MPP with hypoxia, RMPP, and GMPP groups were summarized in Tables 2 and 3. In MPP with hypoxia patients, the average of white blood cell count (WBC), percentage of peripheral neutrophils(N%), CRP, IL-6, ALT and ferritin (fer) was 10.19 × 10^9^/L,68.81%,51.21 mg/L,69.96 pg/ml, 47.17 U/L and 421.61 ng/L respectively, which were significantly higher than those in other groups (*P* all <0.05). And the level of fibrinogen was lowest in the MPP with hypoxia (3.70 g/L: 4.27 g/L: 4.52 g/L, *p* < 0.05). As for LDH and D-dimer, there were statistical differences only between the MPP with hypoxia and the GMPP (*p* < 0.01), besides PCT was observed the difference only between MPP with hypoxia and RMPP groups (*p* < 0.05), In contrast, lactic acid and AST showed no difference among the three groups (p>0.05).

**Table 2:** Laboratory findings of MPP with hypoxia, RMPP, and GMPP patients

Laboratory information	MPP WITH HYPOXIA(***n*** = 69)	RMPP(***n*** = 86)	GMPP(***n*** = 190)
WBC(× 10^9^/L)	10.19 ± 4.61	8.312 ± 3.286*	8.757 ± 3.795**
N,%	68.81 ± 13.32	62.620 ± 13.670*	60.321 ± 13.249**
CRP, mg/L	51.21 ± 49.59	26.273 ± 29.850*	23.771 ± 29.012**
LDH,IU/L	516.29 ± 221.37	471.92 ± 219.04	414.85 ± 168.83**
PCT,ng/ml	0.41 ± 0.68	0.22 ± 0.23*	0.25 ± 1.31
IL-6,pg/ml	69.96 ± 115.23	32.13 ± 28.33*	21.29 ± 28.16**
La,mmol/l	2.82 ± 1.064	2.94 ± 1.13	3.00 ± 1.11
AST,U/L	48.68 ± 42.27	40.03 ± 29.15	37.41 ± 41.25
ALT,U/L	47.17 ± 62.46	24.55 ± 27.43*	23.15 ± 48.36**
Fer,ng/L	421.61 ± 341.06	230.08 ± 265.68*	150.85 ± 167.70**
Fg,g/l	3.70 ± 1.15	4.27 ± 1.75*	4.52 ± 1.77**
D-D,mg/L	1.94 ± 2.91	1.86 ± 4.41	0.49 ± 1.15**

*WBC* White blood cell, *N* Peripheral neutrophils, *CRP* C-reactive protein, *LDH* Lactic dehydrogenase, *PCT* Procalcitonin, *IL-6* Interleukin (IL)-6, *La* Lactic acid, *AST* Aspartate aminotransferase, *ALT* Alanine aminotransferase, *Fer* Ferritin, *Fg* Fibrinogen, *D-D* D-dimer

*MPP WITH HYPOXIA vs RMPP *P* < 0.05, **MPP with hypoxia vs GMPP *P* < 0.05 data are represented by mean ± SD

**Table 3:** Imaging of MPP with hypoxia, RMPP, and GMPP patients

Radiological features	MPP WITH HYPOXIA(***n*** = 69)	RMPP(***n*** = 86)	GMPP(***n*** = 190)	***P***-value
Pulmonary consolidation	55 (79.71%)	69 (80.23%)	123 (64.74%)	0.008
Lobar atelectasis	22 (31.88%)	20 (23.25%)	23 (12.11%)	0.000
Pleural thickening	35 (50.72%)	46 (53.49%)	120 (63.15%)	0.117
Pleural effusion	45 (65.22%)	28 (32.56%)	18 (9.47%)	0.000

Data are presented as number (percentage)

In addition to laboratory findings, the radiological abnormalities were more severe in the MPP with hypoxia. The proportion of pulmonary consolidation among MPP with hypoxia, RMPP, and GMPP was 79.71, 80.23and 64.74% respectively (*p* < 0.01). Pulmonary complications were more likely to occur in the MPP with hypoxia. And there were significant differences among the three groups, including atelectasis (31.88%: 23.25%: 12.11%, *P* < 0.01) and pleural effusion (65.22%%: 32.56%%: 9.47%%, *P* < 0.01). However, the incidence of pleural thickening among the three groups was no statistical difference (*p* > 0.05).

### Clinical course and treatment of patients

Regarding the clinical course, the median duration of fever was 12 (9–14) days in the MPP with hypoxia group, 10 (8–12) days in the RMPP group, and 9 (8–10) days in the GMPP group (*P* < 0. 01). And The median length of hospital stay was 12 (9–15) days in the MPP with hypoxia group, 9 (8–10) days in the RMPP group, and 6 (5–7) days in the GMPP group (*P* < 0. 01). A total of 205 patients (57.90%) were treated with glucocorticoid after admission, and the proportion in the MPP with hypoxia group was significantly higher than that in the other two groups (100% versus 84.06,71.05% *P* < 0 01). Fiberoptic bronchoscopy was performed in 201 cases (56.77%). The number of patients using the fiberoptic bronchoscope in the MPP with the hypoxia group was significantly higher (84.06% vs 70.93% vs 43.16% *P* < 0.01). Moreover, the MPP with the hypoxia group received a higher proportion of oxygen therapy and gamma globulin compared to the other two groups (*P* < 0.01). All patients were treated with azithromycin. In our study, all the children recovered and discharged from the hospital without death.

### Predictive values of the independent correlation factors in patients with MPP with hypoxia

**Fig. 1:**
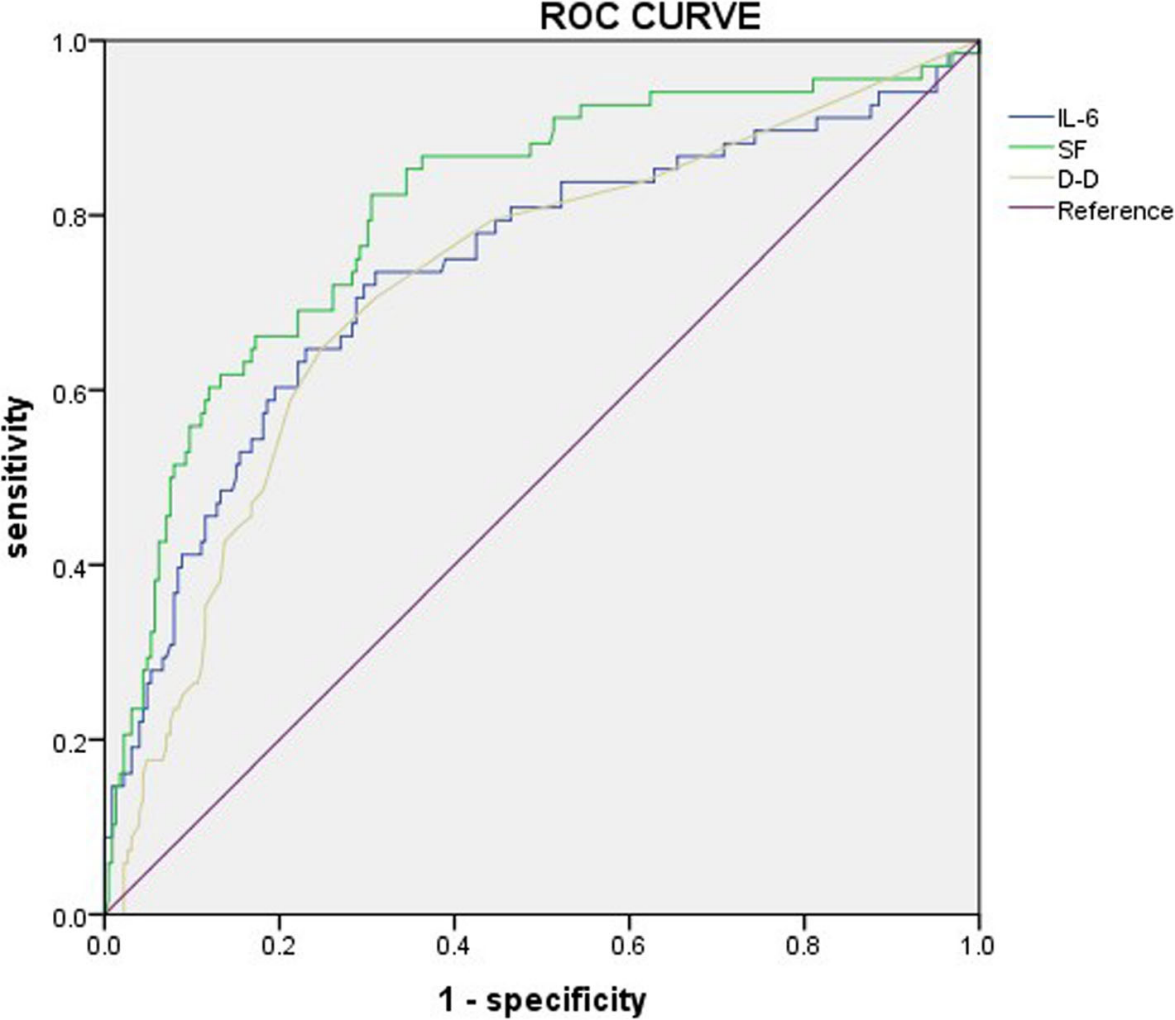
ROC Curve for predictive values of the independent correlation factors in patients with MPP with hypoxia

The ROC analysis was used to explore predictive values of laboratory date for MPP with hypoxia, and the critical value with maximum sensitivity and specificity was also determined in Fig. 1. ROC analysis showed that IL-6, ferritin, and D-dimer were of great significance in the diagnosis of MPP with hypoxia, the area of which under the curve was above 0.7. When the cut-off value for the IL-6, ferritin, and D-dimer was set at 25.47 pg/ml, 171.15 ng/mL, and 0.45 μg/L, the sensitivity and specificity in recognized MPP with hypoxia were 73.5 and 68.9%, 82.4 and 69.3%, 64.7, and 75.1%, respectively in Table 4.

**Table 4:** Predictive values of the independent correlation factors for MPP with hypoxia

Independent factors	Cutoff value	Sensitivity	Specificity	AUC	***P***-value
IL-6,pg/ml	25.47	0.735	0.689	0.737	0.000
Fer,ng/L	174.15	0.824	0.693	0.806	0.000
D-D,mg/L	0.450	0.647	0.751	0.720	0.000

*AUC* Area under the ROC curve, *Cut-off value* The value on the ROC curve is closest to the upper right to take maximum sensitivity and specificity, *P-value* The AUC value of the independent factors compared to ROC curve reference value 0.5. the AUC value of the independent factors compared to the ROC curve reference value 0.5

### Multiple logistic regression analysis for the related factors predicting the MPP with hypoxia

To further evaluate the predictors associated with MPP with hypoxia, multiple logistic regression was performed. IL-6 > 25.47 pg/ml, ferritin > 174.15 ng/mL, and pleural effusion played a significant role in predicting the MPP with hypoxia, with the odds ratio (OR) values of 3.005,3.430, and 3.183, respectively in Table 5.

**Table 5:** Stepwise logistic regression analysis for the related factors predicting the MPP with hypoxia

Variable	B	S.E.	Wald	*P*-value	OR	95%CI	
						Lower	Upper
IL-6,pg/ml	1.100	0.366	9.043	0.003	3.005	1.467	6.156
Fer,ng/L	1.233	0.409	9.066	0.003	3.430	1.538	7.653
Pleural effusion	1.158	0.383	9.122	0.003	3.183	1.502	6.749

## Discussion

Mycoplasma pneumoniae pneumonia continues to be a vital cause of childhood community-acquired pneumonia and is usually a benign self-limited disease. However, sometimes it develops into severe or fulminant cases, endanger the lives [11]. And death is always associated with diffuse pneumonia, acute respiratory distress syndrome (ARDS), brain herniation, vascular thrombosis, and disseminated intravascular coagulation [12–16]. Hypoxia is an important indicator of disease severity. So it is crucial to early diagnosis and early intervention for MPP with hypoxia. However, there were still few studies on MPP with hypoxia, especially in children. So we conducted a retrospective study to identify the associated risk factors of hypoxia in MPP, including 69 cases of the MPP with hypoxia group, and randomly selected 86 cases of the RMPP group and 190 cases of the GMPP group as a control. All cases met the diagnostic criteria.

First of all, there was no difference in age and sex between the three groups. And the median age of all groups was 6 years old, which was consistent with the age of the high incidence of MPP [1].

Secondly, the signs and symptoms in the MPP with hypoxia group were more serious, and the incidence of extrapulmonary complications was higher. In the study, the median time to hypoxia was 10(9–12) days, which was similar to the study on fulminant MPP of Izumikawa et al. [7] Some literature has shown that liver function damage was the most common extrapulmonary complication of MPP with hypoxia [17, 18]. In our research, 13 cases (18.84%) of MPP with hypoxia complicated with liver function damage. Moreover, *MP* infection might contribute to hypercoagulability and cause thromboembolism itself, which was serious extrapulmonary complication [19]. In this study, a total of 7 patients developed thromboembolism, which was located in the lower limb artery (2 cases), lung (4 cases), and heart (1 case). These serious complications also led to longer hospitalization and more complex treatments in the MPP patients with hypoxia. Our study showed the number of people using glucocorticoids in the MPP with hypoxia was more than that in the other two groups, besides only the MPP with hypoxia used gamma immunoglobulin.

At present, there were few studies on MPP with hypoxia. The current theory of excessive immune response causing MPP progression was generally accepted [20–23]. In the laboratory indicators, the level of WBC, neutrophil ratio, CRP, LDH, IL-6, and ferritin were related to MPP with hypoxia, which was similar to the previous case reports [24–26]. Taken together, the evidence suggested a serious immune-inflammatory reaction in the MPP with hypoxia.

The radiological manifestations of MPP were various, mostly bronchial wall thickening, centrilobular nodules, ground-glass attenuation, and consolidation [27]. And our study showed that the imaging findings of MPP with hypoxia were not specific, mainly pulmonary inflammatory consolidation (79.71%). However, MPP with hypoxia was more likely to be accompanied by atelectasis, pleural effusion, and aggravated in a short period. Miyashita et al. indicated that bilateral infiltrates and pleural effusion commonly present in the MPP with hypoxia [18]. It further suggested the severity of the disease, which may be related to the direct invasion of MP and excessive host immune response.

As for the treatment of macrolides, there was no significant difference between the three groups, which may be connected with the high rate of macrolide-resistant M. pneumoniae (MRMP) in China, ranging from 69 to 100% in recent years [28]. Chen Z et al. found that there was no significant difference in the resistance rate of MP between the GMPP group and the RMPP group [29]. But there were reports of severe cases in MRMP among children treated with macrolides [30–32], which may be related to a higher host immune response caused by higher and more persistent stimulation of M. pneumoniae. So, we think a high macrolide-resistant rate may be a factor leading to hypoxia in MPP.

*MP* infection may cause varying degrees of respiratory mucus thrombus obstruction, even form bronchial molding, resulting in airway stenosis and occlusion [33]. Our study showed the MPP with hypoxia group had a higher incidence of mucous plugging. Hence we suspect that may be a cause of hypoxia in MPP. But it still needs further research. Pediatric flexible fiberoptic bronchoscopy can clear respiratory secretions under direct view, relieve airway obstruction, and reduce the occurrence of complications [9]. In our study, the indications for bronchoscopy were atelectasis or segmental inflammatory consolidation on imaging, with lesion area of one or more lung segments, rinsing local lesions, and taking alveolar lavage fluid for pathogen detection [9]. A total of 201 children (58.26%) received fiberoptic bronchoscopy intervention therapy, among which the MPP with hypoxia group received more of this treatment(*p* < 0.01).

However, the use of pediatric flexible fiberoptic bronchoscopy is still controversial. Although we think that the early application of fiberoptic bronchoscopy will shorten the course of the disease and accelerate the recovery of the disease. After all, it is an invasive therapy and blindly expanding the indication may cause harm to the patients. Therefore, in this setting, we should more carefully evaluate before operation and weigh the pros and cons. Besides, the surgeon must have skilled operation skills. It is necessary for the surgeon to observe the situation of the patients closely during and after the operation, and to deal with possible complications in time.

To explore the related risk factors predicting MPP with hypoxia, we used the ROC curve and multivariate logistic regression analysis. ROC analysis revealed that the area under the curve of ferritin, IL-6, and D-dimer were above 0.7, which were helpful to recognize the patients in MPP with hypoxia. And the optimal cutoff value for three factors was 174.15 ng/mL, 25.47 pg/ml, and 0.45 μg/L, respectively. Besides, multiple logistic regression analysis was made to improve the predicted accuracy. We found that ferritin > 174.15 ng/mL, IL-6 > 25.47 pg/ml and pleural effusion were good predictors of MPP with hypoxia. Ferritin represents not only iron reserves but also an inflammatory marker [34]. When inflammation occurs, inflammatory factors act on the body to increase the production of ferritin in serum. At the same time, inflammatory factors cause degeneration and necrosis of local tissue cells, dissolution, and rupture of the cell membrane, resulting in leakage of serum ferritin from damaged cells. As a result, ferritin is significantly increased in the inflammatory response. However, there is still no report about the correlation of ferritin in MPP with hypoxia. Some studies [35] on RMPP reported when the ferritin level was 230 ng/mL or higher, the sensitivity and specificity for diagnosing refractory *MP* pneumonia were 67 and 67%, respectively. In our study, the optimal cutoff point for ferritin was 174.15 ng/mL, with a sensitivity of 82.4% and specificity of 69.3%, and the odds ratio of logistic regression analysis was 3.430. The reason for its difference may be the unrecognized mixed infection in our case. IL-6 plays an important role in the early stage of the immune response. In our research, the area under the curve for IL-6 was 0.737, and the optimal cutoff point was 25.47 pg/ml, with a sensitivity of 73.5% and specificity of 68.9%, the odds ratio of logistic regression analysis was 3.005. Chen et al. showed that the cutoff value of IL-6 for RMPP was 14.75 pg/ml [21]. At present, it is considered that the increase of IL-6 is connected to the severity and course of the disease [36], which further suggests that there may be an excessive immune response in MPP with hypoxia. The advantage of the study is that we first explore the predictors of hypoxia in MPP. Starting from the actual clinical cases, the differences of MPP with hypoxia, RMPP, and GMPP in large samples are compared and analyzed, and the interference of mixed factors is eliminated. It provides a strong basis for the early identification of MPP with hypoxia and has a certain degree of innovation and practicality.

There are several limitations to this study. Firstly, it was a retrospective study, and there may have been some selection bias. Secondly, there may be the presence of mixed infection in some cases which cannot be detected. Therefore, In the future work, we should further carry out long-term multicenter, large sample prospective studies, and further explore the problems found in clinical work, to provide a reliable theoretical basis for early identification, early diagnosis and early intervention of MPP with hypoxia.

## Conclusion

Our study shows that excessive immunological inflammation may play an important role in MPP with hypoxia. FER > 174.15 ng/mL, IL-6 > 25.47 pg/ml and pleural effusion were high risk factors for MPP with hypoxia. MPP with hypoxia patients may need to require glucocorticoid therapy and bronchoscopy.

## Data Availability

The datasets used and/or analysed during the current study are available from the corresponding author on reasonable request.

## Abbreviations

MPP: Mycoplasma pneumoniae pneumonia
RMPP: Refractory Mycoplasma pneumoniae pneumonia;
GMPP: General Mycoplasma pneumoniae pneumonia
MRMP: Macrolide-resistant M. pneumoniae
PCR: Polymerase chain reaction
CRP: C-reactive protein
LDH: Lactic dehydrogenase
PCT: Procalcitonin
IL: Interleukin lactic acid
Fer: Ferritin
Fg: Fibrinogen
ALT: Alanine aminotransferase
AST: Aspartate aminotransferase
ROC: Receiver operating characteristic
